# Spread of Multidrug-Resistant *Rhodococcus equi,* United States

**DOI:** 10.3201/eid2702.203030

**Published:** 2021-02

**Authors:** Sonsiray Álvarez-Narváez, Steeve Giguère, Noah Cohen, Nathan Slovis, José A. Vázquez-Boland

**Affiliations:** University of Georgia, Athens, Georgia, USA (S. Álvarez-Narváez, S. Giguère);; Texas A&M University, College Station, Texas, USA (N. Cohen);; Hagyard Equine Medical Institute, Lexington, Kentucky, USA (N. Slovis);; University of Edinburgh, Edinburgh, Scotland, UK (J.A. Vázquez-Boland)

**Keywords:** multidrug-resistant *Rhodococcus equi*, bacteria, MDR *R. equi*, *erm*(46), pRErm46, Tn*RErm46*, macrolide resistance, rifampin resistance, zoonoses, antimicrobial resistance, bacteria, bacterial infection, United States

## Abstract

Multidrug resistance has been detected in the animal and zoonotic human pathogen *Rhodococcus equi* after mass macrolide/rifampin antibioprophylaxis in endemically affected equine farms in the United States. Multidrug-resistant (MDR) *R. equi* emerged upon acquisition of pRERm46, a conjugative plasmid conferring resistance to macrolides, lincosamides, streptogramins, and, as we describe, tetracycline. Phylogenomic analyses indicate that the increasing prevalence of MDR *R. equi* since it was first documented in 2002 is caused by a clone, *R. equi* 2287, attributable to coselection of pRErm46 with a chromosomal *rpoB*^S531F^ mutation driven by macrolide/rifampin therapy. pRErm46 spillover to other *R. equi* genotypes has given rise to a novel MDR clone, G2016, associated with a distinct *rpoB*^S531Y^ mutation. Our findings illustrate that overuse of antimicrobial prophylaxis in animals can generate MDR pathogens with zoonotic potential. MDR *R. equi* and pRErm46-mediated resistance are currently disseminating in the United States and are likely to spread internationally through horse movements.

*Rhodococcus equi* is a soilborne facultative intracellular actinobacterium that causes pyogranulomatous infections in multiple animal species, including humans. Rhodococcal infection is particularly severe in young foals and immunocompromised persons, in whom it typically manifests as a life-threatening purulent bronchopneumonic disease (*1*–*3*). *R. equi* is able to colonize equids, pigs, and ruminants through 3 different host-specific virulence plasmid types (designated pVAPA, pVAPB, and pVAPN) (*4*). Analysis of the virulence plasmids carried by the isolates and comparison of genomic profiles indicate that human *R. equi* infections originate from animals (*4*–*6*).

*R. equi* is highly prevalent in horse-breeding farms worldwide (*7*). For decades, the standard treatment for *R. equi* pneumonia in foals has been a combination macrolide/rifampin therapy (*8*). In the absence of effective preventive methods, many horse-breeding farms rely on early ultrasonographic detection of infected foals and initiation of macrolide/rifampin prophylaxis before clinical manifestation of the disease (*9*). In the United States, where foal rhodococcosis is often endemic, implementation of this practice has been linked to the emergence of dual macrolide/rifampin–resistant (MR^R^) *R. equi* (*10*–*12*). First detected in the late 1990s, *R. equi* MR^R^ isolates are increasingly prevalent (*11*–*14*), posing a substantial problem because no clinically proven therapeutic alternative is currently available for the treatment of affected foals (*8*). The MR^R^ isolates also represent a potential hazard to human health because of the risk for zoonotic transmission.

We recently determined that the emerging MR^R^ phenotype among *R. equi* equine isolates was linked to a novel methyltransferase gene, *erm*(46), which confers cross-resistance to macrolides, lincosamides, and streptogramins (MLS^R^ phenotype) (*13*). *erm*(46) is part of a 6.9-kb transposable element, Tn*RErm46*, which is carried by the conjugative resistance plasmid pRErm46 (*15*). Upon pRErm46 acquisition, Tn*RErm46* stabilizes itself in *R. equi* by transposing to the host genome, including the conjugative virulence plasmid pVAPA. Despite its high potential for horizontal spread, we found that pRErm46/Tn*RErm46* was restricted to a specific *R. equi* clone, designated 2287, likely because of co-selection with a chromosomal rifampin-resistance *rpoB*^S531F^ mutation in response to macrolide/rifampin therapy (*15*).

We identified the multidrug-resistant (MDR) *R. equi* 2287 clone by analyzing isolates collected during 2002–2011 (*15*). Here, we investigate the spread of the *erm*(46) determinant in a contemporary sample of macrolide-resistant isolates and horizontal spread of pRErm46/TnRErm46, leading to emergence of a further MDR *R. equi* clone associated with a novel *rpoB*^S531Y^ mutation.

## Materials and Methods

### Bacteria

We sequenced the genomes of a random selection of 30 macrolide-resistant and 18 macrolide-susceptible *R. equi* equine clinical strains recovered from pneumonic foals in 5 US states (Florida, Kentucky, Louisiana, New York, and Texas) during 2012–2017 (Appendix Table 1). Whenever possible, at least 1 strain from each category was chosen for each year and US state. The strains from Louisiana were a random collection of 10 convenience-sampled isolates from a single farm. All strains were routinely grown in brain-heart infusion medium (BD, https://www.bd.com) for 48 h at 37°C. Detection of the *erm*(46) gene by PCR was performed as previously described (*13*,*15*).

### Antimicrobial Susceptibility Testing

Susceptibility tests were performed at the Hagyard Equine Medical Institute diagnostic laboratory (Lexington, Kentucky, USA), Texas A&M Veterinary Medical Diagnostic Laboratory (College Station, Texas, USA), and University of Georgia Veterinary Diagnostic Laboratory (Athens, Georgia, USA) according to Clinical and Laboratory Standards Institute (CLSI) guidelines (https://clsi.org). In the absence of specific disk susceptibility interpretive criteria for *R. equi*, CLSI guidelines for *Staphylococcus aureus* were used in accordance with routine practices in veterinary diagnostic laboratories (*11*,*16*). MICs were determined in tryptone soy agar medium by using Etest strips (bioMérieux, https://www.biomerieux.com) according to the manufacturer’s recommendations (*16*). *Staphylococcus aureus* ATCC 29213 was used as a control in all susceptibility tests.

### Genome Sequencing and Phylogenetic Analysis

We extracted bacterial genomic DNA by using DNeasy UltraClean Microbial Kit (QIAGEN, https://www.qiagen.com) following the manufacturer’s instructions. DNA quality (optical density 260/280, ratio 1.8:2) and concentration (>1 µg) of each gDNA sample were verified by using a NanoDrop apparatus (Thermo Fisher Scientific, https://www.thermofisher.com). Single-molecule real-time long-read DNA sequencing was performed at Duke Center for Genomic and Computational Biology (Duke University, Durham, North Carolina, USA). SMRTbell Template Prep Kit 2.0 was used for library preparation of 4–6-kb insert for 8 multiplexed bacterial samples. Samples were run on a PacBio Sequel II system (Pacific Bioscience, https://www.pacb.com). Genomes were assembled de novo by using Canu version 1.9 (*17*). Whole-genome phylogenetic analysis was performed with ParSNP in the Harvest suite, designed for single-nucleotide polymorphism analysis between closely related species or strains (>97% average nucleotide identity) (*18*). The program uses FastTree 2 (*19*) to build approximately maximum-likelihood trees from core-genome single-nucleotide polymorphisms. Trees were visualized in FigTree 1.4.4 (http://tree.bio.ed.ac.uk/software/figtree). Principal component analysis was performed by feeding VCF files extracted from ParSNP alignments to ggfortify package in R software version 3.6.1 (https://cran.r-project.org/web/packages/ ggfortify/index.html). 

### Statistical Analysis

Statistical significance of tetracycline susceptibility data was determined by χ^2^ test and Student t-test. All tests were conducted using Prism software version 8 (https://www.graphpad.com).

## Results

**Figure 1:**
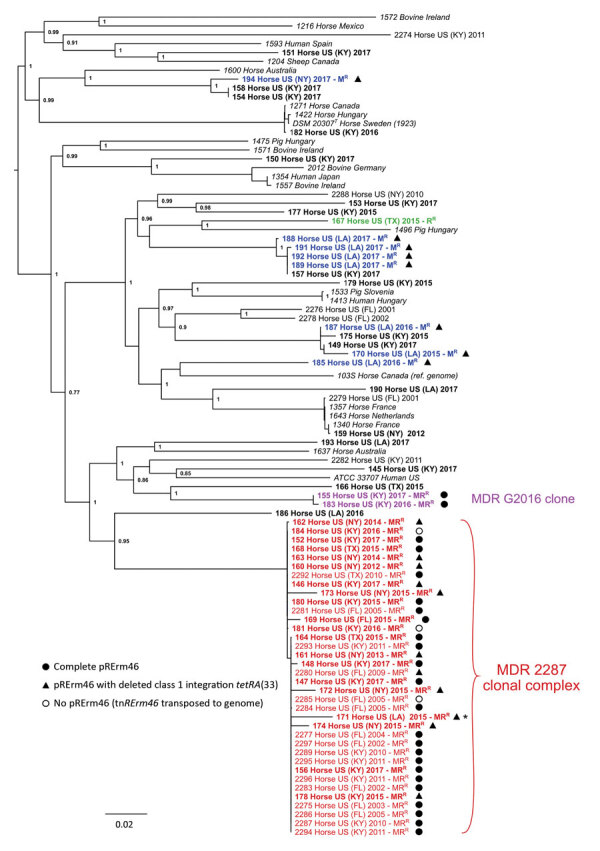
Spread and phylogenetic relationships of MDR *Rhodococcus equi*, United States. Phylogenetic tree of 93 *R. equi* isolates based on core-genome single-nucleotide polymorphism analysis by using ParSNP (*18*). The genomes analyzed are from 58 *erm*(46)-positive M^R^ isolates, 24 control-susceptible isolates from same period and geographic origins, and 23 isolates representative of the genomic diversity of *R. equi*, including the reference genome 103S (*33*) and the type strain DSM 20307^T^ (Appendix Table 1). Tip labels show year of collection and resistance phenotype for the 2001–2017 equine clinical isolates analyzed (the 50 genomes determined in this study are shown in bold, and other genomes are from previous study [*15*]). Red indicates MDR 2287 clonal complex, violet indicates novel MDR G2016 clone, blue indicates genetically diverse M^R^ isolates recovered from a farm in Louisiana during 2015–2017 (MDR 2287 isolate from which they likely acquired the pRErm46 plasmid is indicated by an asterisk), and green indicates an R^R^ isolate (*rpoB* S531K mutation). pRErm46 carriage status is indicated by symbols. Tree graph constructed with FigTree (http://tree.bio.ed.ac.uk/software/figtree). MDR, multidrug-resistant; M^R^, macrolide-resistant; MR^R^, dual macrolide/rifampin resistant; R^R^, rifampin-resistant.

The 30 macrolide-resistant *R. equi* genome sequences determined in this study were subjected to phylogenetic analysis alongside a sample of 18 susceptible isolates from the same period and geographic origins to examine their relationships. The macrolide-resistant isolates had previously tested positive to *erm*(46) by PCR and most (n = 22, 73%) were also resistant to rifampin (MR^R^ phenotype). Of note, 8 of the 2012–2017 *R. equi* isolates examined here were macrolide-only–resistant (Appendix Table 1); to date, dual MR^R^ resistance had been invariably observed (*10*,*11*,*13*,*15*). We also included in our analysis Illumina whole-genome assemblies from 22 equine isolates characterized in our earlier study (n = 16 belonging to the 2287 clone, n = 6 control susceptible isolates) and 23 macrolide-susceptible strains representative of the global genomic diversity of *R. equi* (*20*). Figure 1 shows the core-genome phylogeny of the 93 *R. equi* strains.

### Clonal Spread of MDR *R. equi* 2287

**Figure 2:**
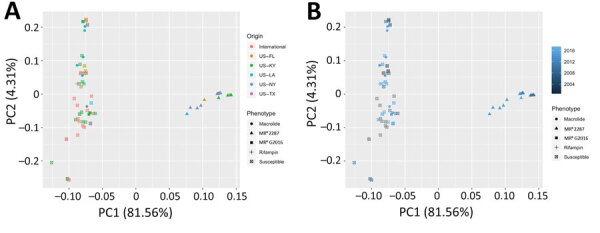
Lack of spatial-temporal circumscription of multidrug-resistant *Rhodococcus equi* clone 2287, United States. Principal component analysis plot was constructed on the basis of the single-nucleotide polymorphism variant calls obtained in the phylogenetic analysis. Isolates are identified by resistance group and color-coded by geographic origin (A) or year of isolation (B). MR^R^, dual macrolide/rifampin resistant; PC1, principal component 1; PC2, principal component 2*.*

Of 22 in total, 20 (91%) of the new MR^R^ isolates clustered together at short genetic distances with the previously characterized MDR 2287 isolates, indicating they correspond to the same clonal population (Figure 1). Accordingly, all of the newly sequenced MR^R^ strains possessed the *rpoB*^S531F^ mutation unique to the 2287 clone. Of those, 2 had lost the pRErm46 plasmid and only carried the Tn*RErm46* transposon (Figure 1), as previously observed in 1 of the 18 isolates from the 2002–2011 series (*15*). Collection times and locations encompassed the entire 2012–2017 period and the 5 US states for the MDR 2287 clonal population. The lack of spatial-temporal circumscription of MDR 2287 in the analyzed sample is illustrated by a principal components analysis in which the only grouping factor for the 93 *R. equi* isolates included in this study is the genetic background of the 2287 clone (Figure 2).

**Figure 3:**
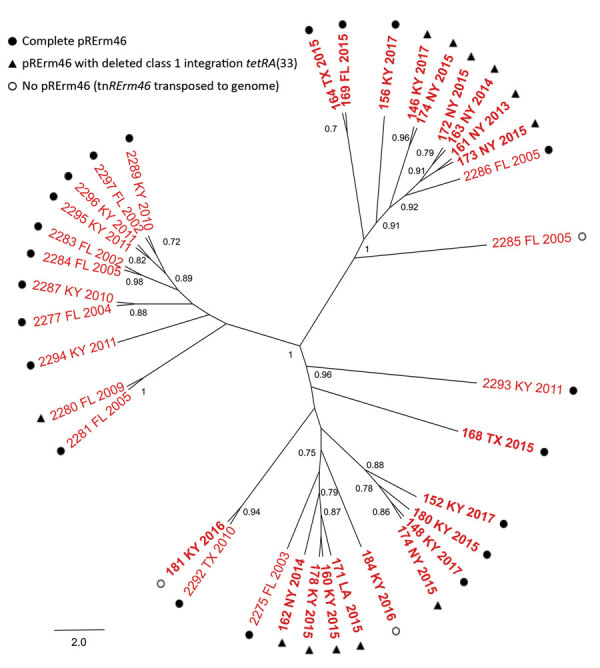
Phylogenetic population structure of multidrug-resistant *Rhodococcus equi* clonal complex 2287, United States. ParSNP core-genome tree of multidrug-resistant 2287 isolates shown in Figure 1. Nodes indicate bootstrap support for 1,000 replicates (values >0.7 shown). Tip labels indicate strain name, source (US state), and year of isolation.

We repeated the phylogenomic analysis with the 36 *R. equi* 2287 sequences from 2002–2017 to assess the microevolution of the clone. This analysis revealed that MDR 2287 had diversified into 3 major radiations (Figure 3), consistent with the clonal structure of *R. equi* evolution (*20*). Of note, 1 of these subclades gathered 11 of the 16 older isolates from 2002–2011, all originating from Florida or Kentucky. The remaining 5 older isolates were distributed in the 2 other subclades in which strains were grouped independently of year of collection or geographic origin. This distribution suggests a pattern of spread defined by the diversification of MDR 2287 into subclonal lineages and increasing exchange between horse farms of a progressively diverse clonal population.

### Dissemination of pRErm46 and Emergence of Novel *R. equi* MDR Clone

Ten macrolide-resistant isolates also carried pRErm46 but did not belong to the MDR 2287 clone and were genetically diverse. Most appeared as singletons interspersed among the different lines of descent in the *R. equi* tree (Figure 1). In this group, 8 strains corresponded to the previously mentioned macrolide-only–resistant isolates (i.e., rifampin susceptible, no *rpoB* mutation; MIC <0.125–1.25 μg/mL). All but 1 of these isolates originated from the same farm in Louisiana in which an MDR 2287 isolate (no. 171) was recovered during the same period. This circumstance suggests a scenario in which the entry of MDR 2287 into this farm resulted in the conjugal spread of pRErm46 to different members of the heterogeneous *R. equi* populations that are typically found colonizing horse-breeding environments, or even individual animals within the same farm (*21*,*22*).

Of interest, 2 of the non-2287 macrolide-resistant isolates, numbers 155 (recovered in Kentucky in 2017) and 183 (recovered in Kentucky in 2016), were also resistant to rifampin (MIC>32 μg/mL) (Figure 1). These 2 nearly genomically identical MR^R^ strains carried the pRErm46 plasmid and a chromosomal *rpoB* mutation, Ser531Tyr (*Escherichia coli* numbering), distinct from that in MDR 2287 and novel in *R. equi*. Both MR^R^ isolates constitute a new emerging MDR *R. equi* clone, first detected in 2016, which we designated G2016. 

Collectively, these data indicate that the pRErm46 macrolide-resistance plasmid, until now unique to the 2287 clone, has recently undergone horizontal transfer events to multiple *R. equi* genotypes. These transfers gave rise to novel MDR clones when associated with an *rpoB* mutation.

### pRErm46 Variability and Tetracycline Resistance

**Figure 4:**
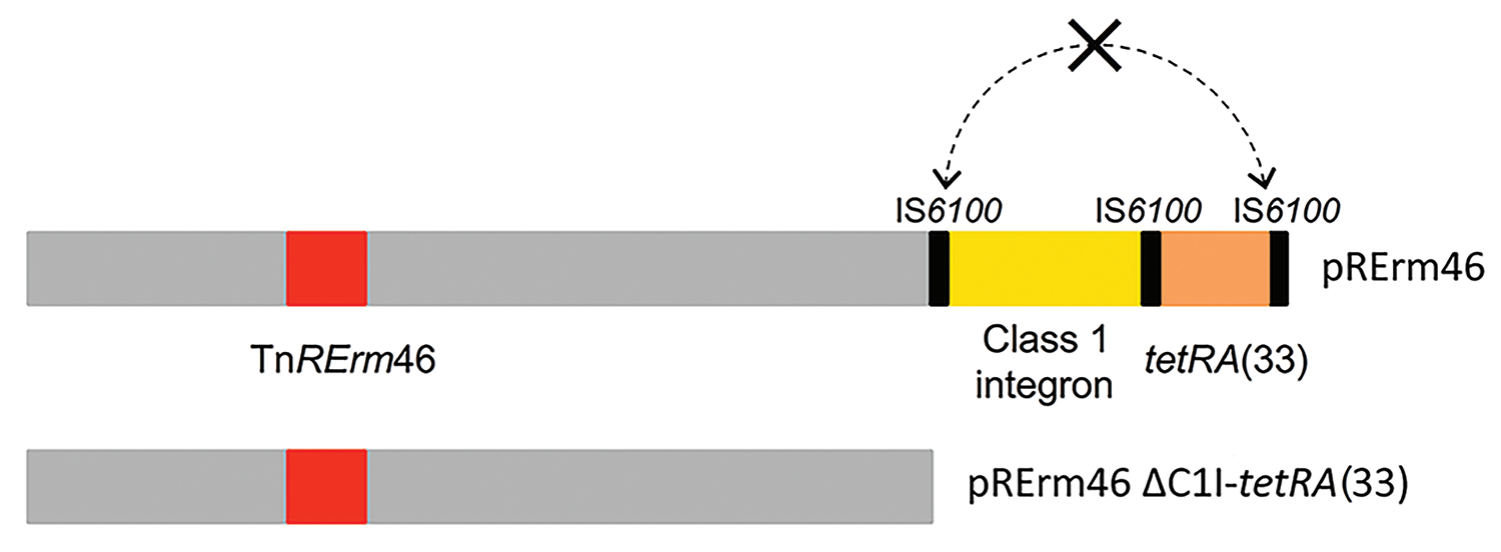
Schematic of ΔC1I*-tetRA*(33) deletion in *Rhodococcus equi* pRErm46 macrolide resistance plasmid. Top bar shows full-size plasmid with the Tn*RErm46* transposon carrying the macrolide-resistance *erm*(46) gene (in red, represented at nt position 32,567 [pRErm46 (PAM 2287) coordinates] common to all pRErm46 plasmids; additional Tn*RErm46* copies generated by transposition from original insertion may be present) and class 1 integron (C1I, in yellow) with associated *tetRA*(33) tetracycline-resistance cassette (peach). Bottom bar shows pRErm46 plasmid with the ΔC1I-*tetRA*(33) deletion. The deletion likely occurs through double crossover between the directly repeated flanking IS6100 sequences (dotted double arrow). Álvarez-Narváez et al. (*15*) includes detailed descriptions of pRErm46 plasmid and Tn*RErm46* transposon.

pRErm46 also harbors a class 1 integron (C1I) with a *tetR*-*tetA* cassette encoding a putative tetracycline efflux pump homologous to TetA(33) from the corynebacterial plasmid pTET3 (*15*,*23*). TetA efflux pumps are often carried by transposons and are one of the most prevalent tetracycline-resistance mechanisms (*24*). Both the C1I and *tetRA* determinant from pRErm46 are virtually identical to those from pTET3, including flanking IS*6100* insertion sequences (*15*). Blast alignments revealed that the C1I-*tetRA*(33) region was deleted in 17 of the 43 (40%) pRErm46 plasmids (Figures 1, 3), presumably because of recombination between the duplicated IS*6100*s (Figure 4). Similar rearrangements have been reported in other integrons carrying directly repeated IS*6100* copies (*25*,*26*). Confirming the predicted functionality of pRErm46’s *tetRA*(33) determinant, pRErm46-positive isolates were resistant to tetracycline, in contrast to those carrying the ΔC1I*-tetRA*(33) form of the resistance plasmid (Table). However, all *R. equi* isolates were susceptible to the semisynthetic tetracycline derivative doxycycline, regardless of pRErm46 plasmid carriage (Table). This finding is consistent with previous data on *Corynebacterium glutamicum* showing that TetA(33) does not confer substantial cross-resistance to doxycycline (*23*).

**Table:** Effect of absence of *tetRA*(33) determinant from pRErm46 plasmid on *R. equi* susceptibility to tetracycline and doxycycline, determined on macrolide-resistant isolates collected during 2012–2017*

Antibiotic	pRErm46			pRErm46 ΔC1I*-tetRA*(33)	
Phenotype†	MIC, μg/mL‡	Phenotype	MIC, μg/mL
Tetracycline	Resistant (100)§	21.33 (8–48)¶		Susceptible (100)§	1.97 (0.38–3)¶
Doxycycline	Susceptible (100)	3.35 (0.75–6)**		Susceptible (100)	1.06 (0.25–3)**

*Susceptibility data to other relevant antimicrobials are shown in Appendix Table 2). †Determined by disk diffusion technique. Isolate percentage shown in parenthesis. Zone diameter susceptibility breakpoints based on Clinical and Laboratory Standards Institute interpretive criteria for *Staphylococcus aureus*, routinely used for *R. equi* susceptibility testing in the absence of specific approved criteria for this species (*11**,**16*).  ‡Minimal inhibitory concentration determined using Etest strips. Mean value (range in parenthesis). §p<0.001 by χ^2^ test. ¶p<0.001 by t-test.  **p<0.001 by t-test. Presence of TetRA(33) appears to induce a small, statistically significant MIC increase, but MIC remains below the Clinical and Laboratory Standards Institute susceptibility breakpoint for doxycycline (susceptible <4 μg/mL, intermediate 8 μg/mL, resistant >16 μg/mL).

Whereas a ΔC1I*-tetRA*(33) plasmid deletion was detected in only 1 of the older (2002–2011) MDR 2287 isolates, the deletion was found in 10 of the 18 pRErm46-positive clonal isolates recovered during 2012–2017 (Figure 1). Deleted pRErm46s are observed in each of the clonal radiations of the MDR 2287 population and coexist with complete plasmids in more basal branches (Figure 3), indicating increasing occurrence because of repeated independent deletion events. The deletion was detected in all of the genetically heterogeneous macrolide-only–resistant *R. equi* isolates and the MDR 2287 (isolate no. 171) recovered from the Louisiana farm during the same period. This finding supports the notion that the latter was the source from which pRErm46 had spread to other locally prevalent *R. equi* genotypes in that particular farm.

## Discussion

This study demonstrates that the increasing prevalence of MR^R^ *R. equi* since its emergence in the late 1990s–early 2000s in equine farms in the United States (*11*–*14*) is primarily caused by the spread of the recently identified MDR 2287 clone (*15*). The oldest characterized MDR 2287 isolate dates from 2002 and was recovered in Kentucky (*15*) (Figure 1), where the clone likely emerged after the implementation of mass macrolide/rifampin antibiotic prophylaxis in foals (*10*). Since then, *R. equi* MDR 2287 has been frequently transferred between geographically distant farms, presumably through carrier horses. Active exchange of *R. equi* populations, previously noted in our earlier study (*20*), is evident in the United States and internationally when considering the phylogenetic tree in Figure 1. For example, the strains recovered from the Louisiana farm in this study are essentially identical to others found elsewhere in the United States. Also, terminal branches of the *R. equi* tree contain nearly identical equine isolates from different countries (e.g., the United States, France, and the Netherlands, or, in another case, Canada, Hungary, Sweden, and the United States) (Figure 1).

Despite the diversity of *R. equi* genotypes that typically circulate in farms (*21*,*22*), the highly horizontally transferable *erm*(46) (Tn*RErm46*) determinant remains largely confined to MDR 2287. This paradoxical clonal restriction is the probable consequence of the simultaneous requirement for *erm*(46) and the *rpoB* mutation under dual macrolide/rifampin pressure. More specifically, the clonal restriction is likely determined by the low odds of pRErm46/Tn*RErm46* and a high-resistance *rpoB* mutation (such as Ser531Phe in MDR 2287 or Ser531Tyr in MDR G2016) being acquired concurrently, and the latter effectively linking the mobile *erm*(46) determinant to a specific chromosomal background (*15*).

This interpretation implies several predictions. First, under dual macrolide/rifampin pressure, spread of an existing MR^R^ strain through horse movements is more likely to contribute to the bulk of resistance than the generation of new MR^R^ strains (*15*). Second, continued macrolide/rifampin therapy might eventually lead to the emergence of new MR^R^ clones, such as G2016 identified in this study, detected in 2016 in Kentucky and characterized by a novel *rpoB*^S531Y^ mutation. Third, and importantly, if dual macrolide/rifampin selection ceases, unrestricted pRErm46/Tn*RErm46* horizontal transfer to other *R. equi* strains might occur. Our data appear to support these 3 possibilities.

The first and second scenarios are expected in horse-breeding areas such as Kentucky, Texas, or Florida, where *R. equi* is endemic and macrolide/rifampin antibiotic prophylaxis has been commonly practiced (*10*,*27*,*28*). Less intensive and more targeted antibiotic therapy is more likely in areas with smaller horse populations such as Louisiana (*29*), where pRErm46 spillover outside the MDR 2287 clone was detected (the third scenario). We hypothesize that a less intensive antibiotic pressure, perhaps involving macrolide monotherapy or a macrolide in combination with non-rifampin antibiotic drugs, disrupted the linkage between *erm*(46) and *rpoB*^S531F^ in the MDR 2287 strain found in the Louisiana farm, enabling the transfer of the plasmid to other locally prevalent *R. equi* strains (Figure 1).

Our analyses show that MDR 2287 has diversified since its first documented isolation into a clonal complex with several radiations (Figure 3). We also detected signs of microevolution in pRErm46, with a substantial rate of deletion of the C1I-*tetRA*(33) region in the 2012–2017 macrolide-resistant *R. equi* cohort, resulting in loss of tetracycline resistance. The clinical significance of this finding is unclear because tetracyclines are not used to treat *R. equi* infections in foals. An exception is doxycycline, which, because of its higher oral bioavailability in foals, greater tissue penetration, and better activity against gram-positive bacteria, might be used in cases of macrolide intolerance (or resistance) (*2*,*8*,*30*). However, our data indicate that the pRErm46-encoded TetA33 does not confer clinically relevant cross-resistance to this semisynthetic tetracycline derivative. Genetic dispensability due to lack of antibiotic selection fitness advantage might therefore be the likely reason for the increasing occurrence of ΔC1I-*tetRA*(33) pRErm46 plasmids in the macrolide-resistant *R. equi* population.

MDR *R. equi* shows resistance to several clinically relevant antibiotic drugs, including macrolides, lincosamides; streptogramins, and, in a substantial proportion, also tetracycline, all conferred by the pRErm46 conjugative plasmid; and rifampin conferred by a chromosomal *rpoB*^S531F/Y^ mutation. MDR *R. equi* also demonstrates intrinsic resistance to chloramphenicol (Appendix Table 2), which is often observed in *R. equi*. All of these antibiotic drugs are listed as critically or highly important for human medicine by the World Health Organization (*31*). Around 9% of human *R. equi* infections are caused by equine-derived (pVAPA-positive) strains, and about half of human cases are caused by porcine-derived (pVAPB-positive) isolates (*5*), which recent in vitro data demonstrate can also acquire pRErm46 (*32*). Therefore, in addition to compromising the therapeutic management of equine *R. equi* infection, these isolates represent a potential hazard to human health because of the risk of zoonotic transmission (or horizontal spread of the pRErm46 resistance plasmid to other pathogens, either directly or through environmental microbiota [*32*]).

Although our study is not systematic and therefore probably underestimates the extent of MDR *R. equi* spread, our results provide valuable insight into the determinants underlying its emergence and dissemination. The data suggest a pattern of MDR *R. equi* spread and evolution directly determined by antibiotic pressure in equine farms. The stable therapeutic regimen applied over years for *R. equi* facilitates a unique understanding of the factors affecting the generation and evolution of MDR clones, and specifically how combination therapy might help in limiting the horizontal transfer of resistance. Although MDR *R. equi* is, to our knowledge, still limited to the equine population in the United States, our data predict a scenario of international spread through horse movements, indicating the need for interventions to control its dissemination and potential zoonotic transmission.

Appendix Additional information on spread of multidrug-resistant *Rhodococcus equi*, United States.
