# Supplementary material for: Spread of Multidrug-Resistant Rhodococcus equi, United States
Source: Emerg Infect Dis. 2021 Feb;27(2):529–37. doi: 10.3201/eid2702.203030 (PMC7853588; doi:10.3201/eid2702.203030)
Supplement: Appendix — Additional information on spread of multidrug-resistant Rhodococcus equi, United States. [file 20-3030-Techapp-s1.pdf]

Article DOI: https://doi.org/10.3201/eid2702.203030

Spread of Multidrug–Resistant *Rhodococcus equi,* United States

Appendix

Appendix Table 1. *R. equi* genomes used in study of multidrug–resistant *Rhodococcus equi*, United States*

Isolate	Host	Isolation year	Origin	Resistance phenotype	Accession No.	Source (reference)
PAM 2274	Horse	2011	Kentucky	S	LWTQ00000000	(*1*)
PAM 2275	Horse	2003	Florida	MR^R	MULU00000000	(*1*)
PAM 2276	Horse	2001	Florida	S	LWTR00000000	(*1*)
PAM 2277	Horse	2004	Florida	MR^R	MUMB00000000	(*1*)
PAM 2278	Horse	2002	Florida	S	MUMA00000000	(*1*)
PAM 2279	Horse	2001	Florida	S	LWTS00000000	(*1*)
PAM 2280	Horse	2009	Florida	MR^R	MULW00000000	(*1*)
PAM 2281	Horse	2005	Florida	MR^R	MULT00000000	(*1*)
PAM 2282	Horse	2011	Kentucky	S	LWTT00000000	(*1*)
PAM 2283	Horse	2002	Florida	MR^R	MULY00000000	(*1*)
PAM 2284	Horse	2005	Florida	MR^R	MULZ00000000	(*1*)
PAM 2285	Horse	2005	Florida	MR^R	LWTU00000000	(*1*)
PAM 2286	Horse	2005	Florida	MR^R	MULX00000000	(*1*)
PAM 2287	Horse	2010	Kentucky	MR^R	LWTV00000000	(*1*)
PAM 2288	Horse	2010	New York	S	LWTW00000000	(*1*)
PAM 2289	Horse	2010	Kentucky	MR^R	MUXK00000000	(*1*)
PAM 2292	Horse	2010	Texas	MR^R	MVDT00000000	(*1*)
PAM 2293	Horse	2011	Kentucky	MR^R	MVDU00000000	(*1*)
PAM 2294	Horse	2011	Kentucky	MR^R	MVDV00000000	(*1*)
PAM 2295	Horse	2011	Kentucky	MR^R	MVDQ00000000	(*1*)
PAM 2296	Horse	2011	Kentucky	MR^R	MVDR00000000	(*1*)
PAM 2297	Horse	2002	Florida	MR^R	MUXJ00000000	(*1*)
CL_s145	Horse	2017	Kentucky	S	SAMN13392178	Current study
CL_mdr146	Horse	2017	Kentucky	MR^R	SAMN13392179	Current study
CL_s147	Horse	2017	Kentucky	MR^R	SAMN13392180	Current study
CL_mdr148	Horse	2017	Kentucky	MR^R	SAMN13392181	Current study
CL_s149	Horse	2017	Kentucky	S	SAMN13392182	Current study
CL_s150	Horse	2017	Kentucky	S	SAMN13392183	Current study
CL_s151	Horse	2017	Kentucky	S	SAMN13392184	Current study
CL_mdr152	Horse	2017	Kentucky	MR^R	SAMN13392185	Current study
CL_s153	Horse	2017	Kentucky	S	SAMN13392186	Current study
CL_s154	Horse	2017	Kentucky	S	SAMN13392187	Current study
CL_mdr155	Horse	2017	Kentucky	MR^R	SAMN13392188	Current study
CL_mdr156	Horse	2017	Kentucky	MR^R	SAMN13392189	Current study
CL_s157	Horse	2017	Kentucky	S	SAMN13392190	Current study
CL_s158	Horse	2017	Kentucky	S	SAMN13392191	Current study
CL_s159	Horse	2012	New York	S	SAMN13392192	Current study
CL_mdr160	Horse	2012	New York	MR^R	SAMN13392193	Current study
CL_mdr161	Horse	2013	New York	MR^R	SAMN13392194	Current study
CL_mdr162	Horse	2014	New York	MR^R	SAMN13392195	Current study
CL_mdr163	Horse	2014	New York	MR^R	SAMN13392196	Current study
CL_mdr164	Horse	2015	Texas	MR^R	SAMN13392197	Current study
CL_s166	Horse	2015	Texas	S	SAMN13392199	Current study
CL_s167	Horse	2015	Texas	R^R	SAMN13392200	Current study
CL_mdr168	Horse	2015	Texas	MR^R	SAMN13392201	Current study
CL_mdr169	Horse	2015	Florida	MR^R	SAMN13392202	Current study
CL_mdr170	Horse	2015	Louisiana	M^R	SAMN13392203	Current study
CL_mdr171	Horse	2015	Louisiana	MR^R	SAMN13392204	Current study
CL_mdr172	Horse	2015	New York	MR^R	SAMN13392205	Current study
CL_mdr173	Horse	2015	New York	MR^R	SAMN13392206	Current study
CL_mdr174	Horse	2015	New York	MR^R	SAMN13392207	Current study
CL_s175	Horse	2015	Kentucky	S	SAMN13392208	Current study
CL_s177	Horse	2015	Kentucky	S	SAMN13392210	Current study

Isolate	Host	Isolation year	Origin	Resistance phenotype	Accession No.	Source (reference)
CL_mdr178	Horse	2015	Kentucky	MR^R	SAMN13392211	Current study
CL_s179	Horse	2015	Kentucky	S	SAMN13392212	Current study
CL_mdr180	Horse	2015	Kentucky	MR^R	SAMN13392213	Current study
CL_mdr181	Horse	2016	Kentucky	MR^R	SAMN13392214	Current study
CL_s182	Horse	2016	Kentucky	S	SAMN13392215	Current study
CL_mdr183	Horse	2016	Kentucky	MR^R	SAMN13392216	Current study
CL_mdr184	Horse	2016	Kentucky	MR^R	SAMN13392217	Current study
CL_mdr185	Horse	2016	Louisiana	M^R	SAMN13392218	Current study
CL_s186	Horse	2016	Louisiana	S	SAMN13392219	Current study
CL_mdr187	Horse	2016	Louisiana	M^R	SAMN13392220	Current study
CL_mdr188	Horse	2017	Louisiana	M^R	SAMN13392221	Current study
CL_mdr189	Horse	2017	Louisiana	M^R	SAMN13392222	Current study
CL_s190	Horse	2017	Louisiana	S	SAMN13392223	Current study
CL_mdr191	Horse	2017	Louisiana	M^R	SAMN13392224	Current study
CL_mdr192	Horse	2017	Louisiana	M^R	SAMN13392225	Current study
CL_s193	Horse	2017	Louisiana	S	SAMN13392226	Current study
CL_mdr194	Horse	2017	New York	M^R	SAMN13392227	Current study
103S	Horse	–	Canada	S	NCBI RefSeq<br>NC_014659.1<br>Genbank<br>FN563149.1	(*2*)
DSM20307^T	Horse	–	Sweden	S	LWTX00000000	(*3*)
ATCC33707	Human	–	Canada	S	NCBI RefSeq<br>NZ_CM001149.1<br>GenBank<br>GCA_000164155.2	–
PAM 1204	Sheep	–	Canada	S	LWBN00000000	(*3*)
PAM 1216	Horse	–	Mexico	S	LWHS00000000	(*3*)
PAM 1271	Horse	–	Canada	S	LWIC00000000	(*3*)
PAM 1340	Horse	–	France	S	LWHT00000000	(*3*)
PAM 1354	Human	–	Japan	S	LWHU00000000	(*3*)
PAM 1357	Horse	–	France	S	LWHV00000000	(*3*)
PAM 1413	Human	–	Hungary	S	LWHW00000000	(*3*)
PAM 1422	Horse	–	Hungary	S	LWHX00000000	(*3*)
PAM 1475	Pig	–	Hungary	S	LWHY00000000	(*3*)
PAM 1496	Pig	–	Hungary	S	LWHZ00000000	(*3*)
PAM 1533	Pig	–	Slovenia	S	LWIA00000000	(*3*)
PAM 1557	Bovine	–	Ireland	S	LWIB00000000	(*3*)
PAM 1571	Bovine	–	Ireland	S	LWTO00000000	(*3*)
PAM 1572	Bovine	–	Ireland	S	LXFI00000000	(*3*)
PAM 1593	Human	–	Spain	S	LXFH00000000	(*3*)
PAM 1600	Horse	–	Australia	S	LXFG00000000	(*3*)
PAM 1637	Horse	–	Australia	S	LWHR00000000	(*3*)
PAM 1643	Horse	–	Netherlands	S	LWTP00000000	(*3*)
PAM 2012	Bovine	–	Germany	S	LWTY00000000	(*3*)

*M^R, macrolide resistant; MR^R, macrolide and rifampin resistant; R^R, rifampin resistant; S, susceptible; –, data not available.

Appendix Table 2. Susceptibility of macrolide–resistant (pRErm46/Tn*RErm46*–carrying) *R. equi* isolates (2012–2017) to clinically relevant antimicrobials in equine medicine*

Antimicrobial	MDR 2287 clone (n = 22)		G2016 clone (n = 2)		Nonclonal isolates (n = 7)	
agent	Phenotype†	MIC µg/mL‡	Phenotype	MIC µg/mL	Phenotype	MIC µg/mL
Ceftiofur	S (95.5)	ND	S (100)	ND	S (100)	ND
Chloramphenicol	R (100)	32.5 (16–64)	R (100)	24 (16–32)	S/R (71.4/28.6)	11.1 (4–32)
Enrofloxacin	S (95.5)	0.39 (0.125–2)	S (100)	0.29 (0.19–0.38)	S (100)	0.22 (0.064–0.38)
Gentamicin	S (100)	0.56 (0.25–1.5)	S (100)	0.37 (0.25–0.5)	S (100)	0.51 (0.25–0.75)
Rifampin	R (100)	≥4–256	R (100)	>32	S (100)	0.38 (0.19–1)

*Typical levels of macrolide resistance: MIC_{90} = 64 µg/ml for chlarithromycin, >256 µg/ml for azithromycin and erythromycin; range ≥4 to >256 µg/ml (*4*). See Table (https://wwwnc.cdc.gov/EID/article/27/2/20–2030–T1.htm) for tetracycline and doxycycline data. ND, not determined; R, resistant; S, susceptible.

†Determined by disk diffusion technique. Isolate percentage shown in parenthesis. Zone diameter susceptibility breakpoints based on Clinical and Laboratory Standards Institute interpretive criteria for *Staphylococcus aureus*, routinely used for *R. equi* susceptibility testing in the absence of specific approved criteria for this species (Table).

‡Minimal inhibitory concentration determined by using Etest strips. Mean value (range in parenthesis).

References

<jrn>1. Álvarez–Narváez S, Giguère S, Anastasi E, Hearn J, Scortti M, Vázquez–Boland JA. Clonal confinement of a highly mobile resistance element driven by combination therapy in *Rhodococcus equi*. MBio. 2019;10:e02260–19. PubMed https://doi.org/10.1128/mBio.02260–19</jrn>

<jrn>2. Letek M, González P, Macarthur I, Rodríguez H, Freeman TC, Valero–Rello A, et al. The genome of a pathogenic R*hodococcus*: cooptive virulence underpinned by key gene acquisitions. PLoS Genet. 2010;6:e1001145. PubMed https://doi.org/10.1371/journal.pgen.1001145</jrn>

<jrn>3. Anastasi E, MacArthur I, Scortti M, Alvarez S, Giguère S, Vázquez–Boland JA. Pangenome and phylogenomic analysis of the pathogenic actinobacterium *Rhodococcus equi*. Genome Biol Evol. 2016;8:3140–8. PubMed https://doi.org/10.1093/gbe/evw222</jrn>

<jrn>4. Anastasi E, Giguère S, Berghaus LJ, Hondalus MK, Willingham–Lane JM, MacArthur I, et al. Novel transferable *erm*(46) determinant responsible for emerging macrolide resistance in *Rhodococcus equi*. J Antimicrob Chemother. 2015;70:3184–90. PubMed</jrn>
